# The role of structural dynamics in the thermal adaptation of hyperthermophilic enzymes

**DOI:** 10.3389/fmolb.2022.981312

**Published:** 2022-09-07

**Authors:** Giuliana Fusco, Francesco Bemporad, Fabrizio Chiti, Christopher M. Dobson, Alfonso De Simone

**Affiliations:** ^1^ Department of Chemistry, University of Cambridge, Cambridge, United Kingdom; ^2^ Section of Biochemistry, Department of Experimental and Clinical Biomedical Sciences “Mario Serio”, University of Florence, Florence, Italy; ^3^ Department of Life Sciences, Imperial College London, London, United Kingdom; ^4^ Department of Pharmacy, University of Naples “Federico II”, Naples, Italy

**Keywords:** thermophilic proteins, protein dynamics, NMR, residual dipolar couplings, restrained MD simulations

## Abstract

Proteins from hyperthermophilic organisms are evolutionary optimised to adopt functional structures and dynamics under conditions in which their mesophilic homologues are generally inactive or unfolded. Understanding the nature of such adaptation is of crucial interest to clarify the underlying mechanisms of biological activity in proteins. Here we measured NMR residual dipolar couplings of a hyperthermophilic acylphosphatase enzyme at 80°C and used these data to generate an accurate structural ensemble representative of its native state. The resulting energy landscape was compared to that obtained for a human homologue at 37°C, and additional NMR experiments were carried out to probe fast (^15^N relaxation) and slow (H/D exchange) backbone dynamics, collectively sampling fluctuations of the two proteins ranging from the nanosecond to the millisecond timescale. The results identified key differences in the strategies for protein-protein and protein-ligand interactions of the two enzymes at the respective physiological temperatures. These include the dynamical behaviour of a β-strand involved in the protection against aberrant protein aggregation and concerted motions of loops involved in substrate binding and catalysis. Taken together these results elucidate the structure-dynamics-function relationship associated with the strategies of thermal adaptation of protein molecules.

## Introduction

The detailed characterization of the structures and dynamics that proteins adopt in solution is of fundamental importance to elucidate the mechanisms by which processes involving their activity occur in the cell. The dynamical behaviour is particularly relevant to allow proteins to access conformations whose properties can vary significantly from the most stable state situated at the minimum of native free energy landscape (Palmer, 2004; Motlagh et al., 2014). Understanding how fluctuations of backbone and side chain atoms in proteins are coupled with biochemical processes governing cellular function and pathology is among the major challenges of modern structural biology. A number of biophysical techniques are available to probe in detail the nature of protein dynamics, including Fourier-transform infrared spectroscopy (FT-IR) (Koziol et al., 2015), Raman spectroscopy (Nibbering et al., 2005), small angle X-ray scattering (SAXS) (Kikhney and Svergun, 2015), hydrogen/deuterium exchange monitored with mass spectrometry (Imamoglu et al., 2020), single molecule assays (Lapointe et al., 2021), and fluorescence resonance energy transfer (FRET) (Schuler and Eaton, 2008). Among the various experimental methods, nuclear magnetic resonance (NMR) plays a critical role for its ability to directly probe protein dynamics on different biologically relevant timescales, ranging from nanoseconds to milliseconds and beyond (Boehr et al., 2006a; Mittermaier and Kay, 2006). A variety of NMR approaches have enabled to elucidate the nature of complex enzymatic motions such as the gating of the proteasome (Religa et al., 2010), enzyme specificity (Byeon et al., 2013), the catalytic cycles of the dihydrofolate reductase (Boehr et al., 2006b) or allosteric codes in kinases (Das et al., 2007; Kim et al., 2017; Walker et al., 2019) and oligomers (VanSchouwen and Melacini, 2016; Toyama and Kay, 2021). NMR has also generated fundamental knowledge in crucial aspects of the dynamics-function relationship in a variety of proteins, including chaperones (Huang et al., 2016; Burmann et al., 2020), pathological protein oligomers (Fawzi et al., 2011; Fusco et al., 2017), transporters through the membrane (Cady et al., 2010; Williams et al., 2013; Weber et al., 2021), cellular signalling (Bhate et al., 2018), protein-membrane interactions (Resende et al., 2009; Fusco et al., 2016), protein-oligonucleotides interactions (Alfano et al., 2004; Noble et al., 2005), intrinsically disordered proteins (Bernado et al., 2005; Mukrasch et al., 2007; Maltsev et al., 2012; Bah et al., 2015; Theillet et al., 2016; Werbeck et al., 2020; Antonschmidt et al., 2021; Deckert et al., 2021) and many others.

Atomic fluctuations are, however, not only important for function but also act as fundamental determinants to preserve the integrity of native states of proteins under physiological conditions (De Simone et al., 2011a). In this context, a fascinating question is how proteins from hyperthermophilic organisms are able to preserve their function (Wolf-Watz et al., 2004; Butterwick and Palmer, 2006) under conditions where their mesophilic counterparts are largely inactive and even unfolded (Zavodszky et al., 1998; Kumar and Nussinov, 2001; Sterner and Liebl, 2001; Vieille and Zeikus, 2001; Stirnemann and Sterpone, 2017). We here used biomolecular NMR and advanced structural refinement to elucidate this key point by characterising at an atomic level protein backbone dynamics of two enzymes from mesophilic and hyperthermophilic organisms. In particular, the human muscle acylphosphatase (mt AcP) and its homologue from Sulfolobus solfataricus (Sso AcP) were studied at the respective physiological temperatures of 37°C and 80°C. Despite sharing limited sequence identity (25%), the two proteins posses the same ferrodoxin-like fold topology from the acylphospatase-like family (Supplementary Figure S1), including secondary structure elements and catalytic residues, and have the same function in their respective native environments despite having significantly different melting temperatures, i.e. 56°C for mt AcP and 100°C for Sso AcP (Corazza et al., 2006). We were able to measure NMR residual dipolar coupling (RDC) at 80°C and used these data to generate an unprecedented structural ensemble of the hyperthermophyilic Sso AcP, and compared its native free energy landscape to that of mt AcP at 37°C. The analysis of the two enzymes at their physiological temperatures also included NMR experiments probing fast (^15^N relaxation) and slow (hydrogen/deuterium exchange - HX) backbone fluctuations, thus generating a holistic map of the enzymes dynamics ranging from nanoseconds to milliseconds (and beyond).

The results identified common traits in the atomic fluctuations of the two AcPs at their native temperatures of 37°C and 80°C, in line with the concept of corresponding states (Zavodszky et al., 1998; Radestock and Gohlke, 2011; Fields et al., 2015). However, key differences were observed with respect to motions associated with substrate binding as well as with the strategies to avoid edge-to-edge native-like aggregation, which is a recurrent problem in the majority of the β-sheet proteins (Richardson et al., 2002). Taken together, the data on protein dynamics of homologous proteins operating at very different temperatures provide key insights into the thermal adaptation and structure-dynamics-function paradigm in the physiological native environments of proteins.

## Materials and methods

### Expression and purification of mt AcP and Sso AcP.

^1^H, ^15^N, ^13^C, labelled mt AcP and Sso AcP were expressed as a GST fusion proteins in the E. coli strain BL21-Gold (DE3) (Invitrogen), grown in minimal medium by using ^15^N- ammonium chloride and ^13^C-glucose. Purification of mAcP and SsoAcP were then performed following previous protocols (Taddei et al., 1996). Cell lysates were purified using a glutathione column (Sigma Aldrich) following GST cleavage with human plasma thrombin (Sigma Aldrich) in TRIS buffer. Eluted proteins were then buffer exchanged into 30 mM ammonium carbonate buffer at pH 8.0 and then lyophilized.

### NMR samples and spectra assignment of mt AcP at 37°C

NMR measurements of mt AcP were performed at 37°C in 30 mM MOPS buffer at pH 7.0, using an NMR spectrometer operating at the ^1^H frequency of 700 MHz. Temperature was carefully calibrated using the ethylene glycol standard (Spees et al., 2012). The protein net charge under these conditions is expected to be +4.9, ensuring high solubility owing to electrostatic repulsion between molecules. NMR samples were prepared by diluting lyophilised protein into 500 μl of 10% deuterated solution up to a protein concentration of 200 μM. Assignments of the mt AcP was retrieved from our previous work (Fusco et al., 2012) and transferred to the current sample conditions. In the original assignment in phosphate buffer, the resonances of all 97 non-proline residues were present in the ^1^H-^15^N HSQC spectra. As our investigation focused on the ligand-free state of the protein at 37°C, fewer amide N-H resonances (i.e. 86) were detectable. This is in line with previous observations of other acylphosphatase proteins, where the ligand-free state was reported to be affected by the broadening of some NMR resonances due to conformational exchange (De Simone et al., 2011a). All peaks in the ^1^H-^15^N HSQC were assigned, with missing resonances localised predominantly in the N-terminal tail and loops of the protein (1–2, 19–21, 23, 25, 42, 70, 88 and 93). All NMR data were processed and analysed by TopSpin (Bruker BioSpin), NMRPipe and Sparky software packages.

### NMR samples and spectra assignment of Sso AcP at 80°C

NMR analyses of Sso AcP were performed at 80°C in 30 mM MES buffer and pH 6.5, which mimics the physiological conditions of the protein, using an NMR spectrometer operating at the ^1^H frequency of 700 MHz. In all experiments, the pH was measured and adjusted at 80°C. At this pH the net charge of the protein is expected to be-0.5. Despite the mild negative charge, Sso AcP samples could be concentrated up to 250 μM without evident aggregation events during the measurements at 80°C. Samples were prepared by diluting the liophylised protein powder in 500 μl of 10% deuterated buffer solution and by adding a drop of mineral oil on top of the water solution in the NMR tube in order to avoid drying effects due to the water evaporation at 80°C. Temperature was carefully calibrated using the ethylene glycol standard (Spees et al., 2012). NMR resonance assignments of Sso AcP were performed at 80°C following the protocol employed in the assignment of mt AcP (Fusco et al., 2012), which is based on a combination of 3D spectra (HNCA, CBCAcoNH, HNCACB, HNCO, HNcaCO and HNHA) in conjunction with the assignment performed at 25°C (BMRB, entry code: 6398) (Corazza et al., 2006). The ^1^H-^15^N HSQC spectrum of the ligand-free Sso AcP at 80°C showed the resonances of 74 out of 89 non-proline residues. Missing peaks belong to residues 12–14, 27–33, 65, 80, 90, 92 and 98. It is worth noting that in our study, the truncated form of Sso AcP was employed (ΔN11), to include only the ferrodoxin-like domain as in mt AcP.

### ^15^N relaxation experiments

^15^N relaxation experiments were performed at 37°C and 80°C for mt AcP and Sso AcP, respectively. R_1_ rates were measured in ^1^H-^15^N HSQC spectra using the BRUKER pulse-program hsqct1etf3gpsi (avance-version), which is optimized for sensitivity enhancement for measuring ^15^N R_1_ relaxation times. Seven T_1_ delays were employed (0.1, 0.2, 0.4, 0.6, 0.8, 1.0 and 1.2 s), with spectra measured with 1.2 s delay showing 10% of the signal of spectra measured with a T_1_ of 0.1 s. Multiple data points were obtained for each T1. Data points were fitted with a single exponential function to provide R_1_ of each amide N-H of the protein backbone.

R_2_ rates were measured using the pulse-program hsqct2etf3gpsi, with the same spectral properties employed for R_1_ measurements. T_2_ delays were set as 0.017, 0.034, 0.051, 0.068, 0.102, 0.153, 0.170, 0.204, 0.238 and 0.289 s, where spectra measured with the maximum delay showed less than 10% of signal intensity compared with those measured at 0.017 s of T_2_ delay. Multiple data points were obtained for each T_2_ delay. Data points were fitted with a single exponential function to provide R_2_ of each amide N-H of the protein backbone.

Heteronuclear NOEs were measured using the BRUKER pulse-program hsqcnoef3gpsi in a phase sensitive mode using Echo/Antiecho-TPPI gradient selection with decoupling during acquisition. The experiment measured simultaneously saturated and unsaturated NOEs signals in interleaved mode, provinding heteronuclear NOEs from the signal ratio of saturated and unsaturated peaks. Model free analysis was performed with the program Modelfree (Mandel et al., 1995). The analysis was made using datasets measured at single ^1^H frequency, which can lead to some limitations in the interpretations of the data.

### Residual dipolar couplings

Residual dipolar couplings were measured for both Sso AcP and mt AcP by orienting the protein with bicelles or polyacrylamide gels (Ottiger and Bax, 1998; Schwalbe et al., 2001). IPAP spectra were measured on the isotropic and anisotropic samples (Ottiger and Bax, 1998). Inorder to measure RDCs of Sso AcP at 80°C, we used stretched polyacrylamide gels, which showed temperature resistance under these conditions by providing water splitting of ∼11Hz for several days. Splitting of the ^2^H signal was recorded before and after the IPAP experiments, to ensure that alignment had remained constant during the course of the NMR experiment.

Two types of acrylamide gels were produced, including neutral and negatively charged gels. The first were obtained by polymerization in chambers of RDC kits of a mixture of 5.11% (w/v) acrylamide, 0.13% (w/v) bis-acrylamide, 0.1% (w/v) ammonium persulfate, 0.0031% (w/v) TEMED and 100 mM Tris-HCl at pH 8.0. The gels were washed twice with water and twice with the final 30 mM MES buffer at pH6.5. After washing, protein was soaked into the gels and subsequently gels were stretched into the NMR tube. In order to avoid drying effects due to the water evaporation from gel matrix, a drop of mineral oil was placed at the top of the gels in before inserting the tube plunge. Negatively charged gels were produced with a similar protocol except for the employment of acrylic acid as the copolymerisation agent in a 1:3 ratio with acrylamide.

Bicelles for RDCs were obtained from a mixture of short and long chained phospholipids (DMPC and DHPC). All phospholipids were purchased as dry powders from Avanti Polar-Lipids, Inc. (Alabaster, AL) and used without further purification. Bicelles were prepared in 10 mM MOPS buffer, pH 7.0, 0.15 mM sodium azide, 93% H2O (HPLC grade, Aldrich), 7% D2O (99.9%, CIL). Uncharged bicelles were prepared using DMPC and DHPC lipids at a total concentration of 15% w/v (150 mg lipid/ml) and a molar ratio q = [DMPC]:[DHPC] of 2.9. Positively charged bicelles were obtained using a mixture of DMPC, DHPC and CTAB at molar ratio of 2.9:1.0:0.2

### H/D exchange

H/D exchange was measured primarily from the attenuation of ^1^H-^15^N HSQC signal upon dissolving liophylised samples in D_2_O. NMR setting was performed using a D_2_O buffer solution prior dissolving the protein to minimize the lag time before the first ^1^H-^15^N HSQC measurement. Spectral width in the indirect dimension of the ^1^H-^15^N HSQC was optimized to measure a full 2D spectrum in ∼2 min k_obs_ were fitted from the signal decay using single exponential curves.

Measurements for mt AcP were performed at pH 7.0 in 30 mM MOPS buffer by using 100% D_2_O whereas for Sso AcP we employed a 30 mM MES buffer at pH 6.5 in 100% D_2_O.

NMR hydrogen exchange data were using this model

$$closed\overset{k_{op}}{\underset{k_{cl}}{⇌}}open\overset{k_{\mathrm{int}}}{\to }exchanged$$ (1)

where “closed” and “open” are the amide protons in conformations resistant and favourable to the H/D exchange, respectively. The observed hydrogen exchange rate is therefore modelled as:

$$k_{obs}=\frac{k_{op}k_{\mathrm{int}}}{k_{cl}+k_{\mathrm{int}}}$$ (2)

where k_cl_ is the closing rate and k_op_ is the opening rate. The intrinsic rate (k_int_), which depends on factors such as pH, temperature, and protein sequence, was computed using an established method (Connelly et al., 1993). In the EX2 limit (k_cl_ >> k_int_) the observed hydrogen exchange is

$$k_{obs}=\frac{k_{op}k_{\mathrm{int}}}{k_{cl}}$$ (3)

leading to an expression of the protection factor P = (k_int_/k_obs_) that is proportional to the ratio of concentrations of open and closed conformations. We checked that the EX2 limit governs the exchange properties of the proton amides for mt AcP and Sso AcP using ESI-MS experiments and gradients of pH at the respective buffer conditions, by means of a protocol previously successfully employed for an homologous AcP protein (De Simone et al., 2011a).

Very fast amide proton exchange prevented the direct observation of the decay curves. For these amide groups, CLEANEX-PM experiments were employed using the initial slope analysis (Hwang et al., 1998) by using short mixing times τ_m_ (0, 10, 15, 20 and 25 ms) in order to ensure that transverse relaxation does not significantly influence peak intensities.

### Ensemble-averaged MD simulations restrained with RDCs

A number of methods to employ residual dipolar couplings (RDCs)(Tjandra and Bax, 1997; Prestegard et al., 2000) for the characterization of the structure and dynamics of proteins have been proposed, including analytical deconvolution (Prestegard et al., 2000), the Gaussian axial fluctuations method (Salmon et al., 2009) restrained molecular dynamics simulations in which the alignment tensor is fitted to the experimental RDCs (Clore and Schwieters, 2004; De Simone et al., 2009) and direct comparison with molecular dynamics simulations (Showalter and Bruschweiler, 2007). In this study we employed RDC to restrain MD simulations following our previous methods development (De Simone et al., 2009; De Simone et al., 2011b; Montalvao et al., 2012; De Simone et al., 2013a; De Simone et al., 2013b; De Simone et al., 2015). In particular ensemble-averaged restraints in molecular dynamics simulations were employed by calculating the RDC tensor from the shape and charge of each individual structure in the ensemble (De Simone et al., 2011b; Montalvao et al., 2012). This method to calculate RDCs from protein structure has been implemented as a restraint in the GROMACS package for MD simulatons (Hess et al., 2008). Our approach includes the averaging over M replicas of the system to enable imposing the experimental restraints as an ensemble property. This approach is particularly efficient in sampling protein ensembles of conformational heterogeneous states because the replica averaging enables populating simultaneously different conformational basins that are present in solution and that contribute to the experimental observable (De Simone et al., 2009; Montalvao et al., 2012).

Restraints are imposed by adding a pseudoenergy term (E^RDC^) to a standard MD force field (E^FF^)

$$E^{\mathrm{Total}}=E^{\mathrm{FF}}+E^{\mathrm{RDC}}$$ (4)

We employed as E^FF^ the AMBER99SB-ILDN, with additional corrections for improving sidechains (Lindorff-Larsen et al., 2010) and backbone (Best et al., 2008) dihedral angles. The pseudoenergy experimental term is given by

$$E^{RDC}=\alpha \sum_{i}{\left(D^{calc}-D^{\exp}\right)}^{2}$$ (5)

Initial equilibration simulations were performed starting from the NMR structures of mt AcP (Motamedi-Shad et al., 2012) and Sso AcP (Corazza et al., 2006), respectively at 310K and 353K, by solvating the proteins in explicit water models of Tip4pEW (Horn et al., 2004). During these equilibrations, the agreement between calculated and experimental RDCs was minimized by gently raising the restraint force constant α (eq.5). Subsequently, a series of 50 cycles of simulated annealing between 310K and 500K (for mt AcP) or 353K and 500K (for Sso AcP) were carried out to sample effectively the conformational space. The restraints were imposed as averages over M replicas of the protein molecule; we employed in this work M = 16 following our previous works (De Simone et al., 2011b; De Simone et al., 2013a; De Simone et al., 2013b; De Simone et al., 2015). Each cycle was carried out for a total of 8 ns by using an integration step of 2 fs. Of the 50 cycles of temperature annealing the first 20 were excluded from the analysis for convergence purposes. To build the ensemble, conformations at each cycle were extracted in the last ns of the simulation, when the system is equilibrated at 310K (mt AcP) or 353K (Sso AcP).

The simulations were performed in the NPT ensemble by weak coupling the pressure and temperature with external baths. Temperature coupling was performed with the v-rescale method (Bussi et al., 2007) using a coupling constant of 0.1 ps. The pressure was kept constant with the Berendsen method (Berendsen et al., 1984), using a coupling constant of 1.0 ps and at a reference pressure of 1 bar. The isotropic compressibility value was set to 4.5 × 10^–5^ bar^−1^. Electrostatic interactions were treated by using the particle mesh Ewald method.

## 3 Results

### Nanosecond dynamics by ^15^N relaxation

**FIGURE 1:**
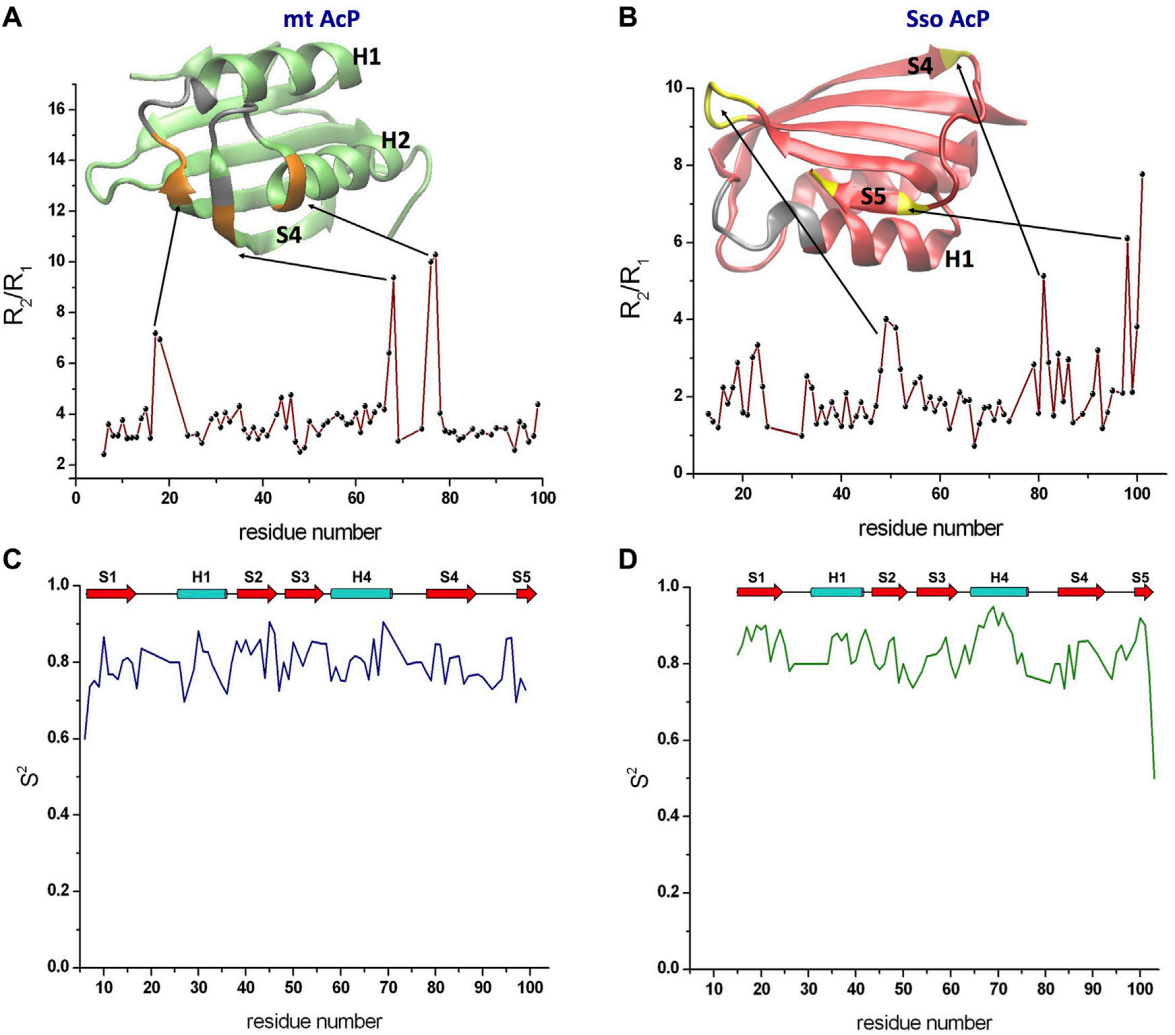
Nanosecond dynamics from ^15^N Relaxation experiments. **(A)** R_2_/R_1_ values along the sequence of mt AcP at 37°C showed a uniform distribution of values for the rigid regions, whereas high ratios identified residues in conformational exchange on slower timescales. The ribbon representation of the protein structure is shown in green, with silver regions indicanting residues whose resonances are broadened beyond detection and orange regions showing residues with high R_2_/R_1_ values. **(B)** R_2_/R_1_ values along the sequence of Sso AcP at 80°C. The protein structure is shown using red ribbons and by coloring in yellow and silver the regions with high R_2_/R_1_ values and resonance broadening, respectively. **(C,D)** Order parameters S^2^ valuesof mt AcP at 37°C **(C)** and Sso AcP at 80°C **(D)** calculated using data from ^15^N relaxation and the model free analysis.

^15^N transverse and longitudinal relaxation rates provided details of nanosecond dynamics of the backbone atoms of mt AcP and Sso AcP (SupplementaryFigure S2). Measurements for mt AcP were performed at 37°C in 30 mM MOPS buffer, pH 7.0, to reproduce the physiological conditions of the native state of this protein. The measured longitudinal relaxation rates R_1_ were found to be tightly distributed around an average of 2.05 ± 0.16 s^−1^ (Supplementary Figure S3). By contrast, transverse relaxation rates R_2_ showed larger variability around an average value of 7.93 ± 2.92 s^−1^. The ratio of these parameters (R_2_/R_1_) was used to gather information on the tumbling rate of the protein as well as to identify possible regions in conformational exchange (Figure 1A). In particular, a uniform distribution of R_2_/R_1_ ratio values was observed in the rigid parts of mt AcP, however, some residues were found to have high ratios that deviate from the values adopted in the rest of the protein, suggesting local conformational exchange on slower timescales. These residues are located in segments flanking regions whose resonances are broadened beyond detection, including residues 17–18 from the loop connecting strand S1 and helix H1 and residues 67–68 and 76–77 located at the extremities of the loop connecting helix H2 and strand S4 (Figure 1A). The spatial proximity to the catalytic site of both residues 17–18 showing high R_2_/R_1_ values and residues whose resonances are completely broadened indicates enhanced local conformational dynamics for the ligand-free form of mt AcP at 37°C.

^15^N transverse and longitudinal relaxation rates of Sso AcP were measured at 80°C in 30 mM MES buffer and pH 6.5 by applying, as all the NMR experiments at this temperature, a drop of mineral oil on top of the solution in order to avoid evaporation/condensation effects within the tube. In contrast with mt AcP, R_1_ values for Sso AcP showed larger variability around an average value of 3.09 ± 0.74 s^−1^. Similar large variability was observed for R_2_ values (average value of 6.28 ± 3.07 s^−1^). R_2_/R_1_ ratio for residues in secondary structure elements were smaller than those found for mt AcP, which is in line with the faster tumbling of Sso AcP at 80°C (Figure 1B). These measurements identified residues showing high R_2_/R_1_ values, indicating slow conformational exchange, however, the location of these regions was different from that mt AcP (Figure 1B). In particular, high R_2_/R_1_ values were found in the loop connecting strands S2 with S3 as well as the C-terminus of strand S4 and both termini of strand S5 (Figure 1B).

Relaxation rates R_2_ and R_1_ were then used in conjunction with hetNOEs in a model free analysis (Lipari and Szabo, 1982) providing the order parameter S^2^ (Figures 1C,D). The data revealed that Sso AcP is generally more rigid at 80°C than mt AcP at 37°C, despite having a significantly higher physiological temperature (average S^2^ values of 0.84 ± 0.05 in Sso AcP and 0.79 ± 0.05 in mt AcP). This observation is specifically evident for secondary structure elements, particularly in the helix H2 where the average S^2^ value is 0.87 ± 0.06 in Sso AcP and 0.80 ± 0.05 in mt AcP.

### Microsecond dynamics by residual dipolar couplings.

**FIGURE 2:**
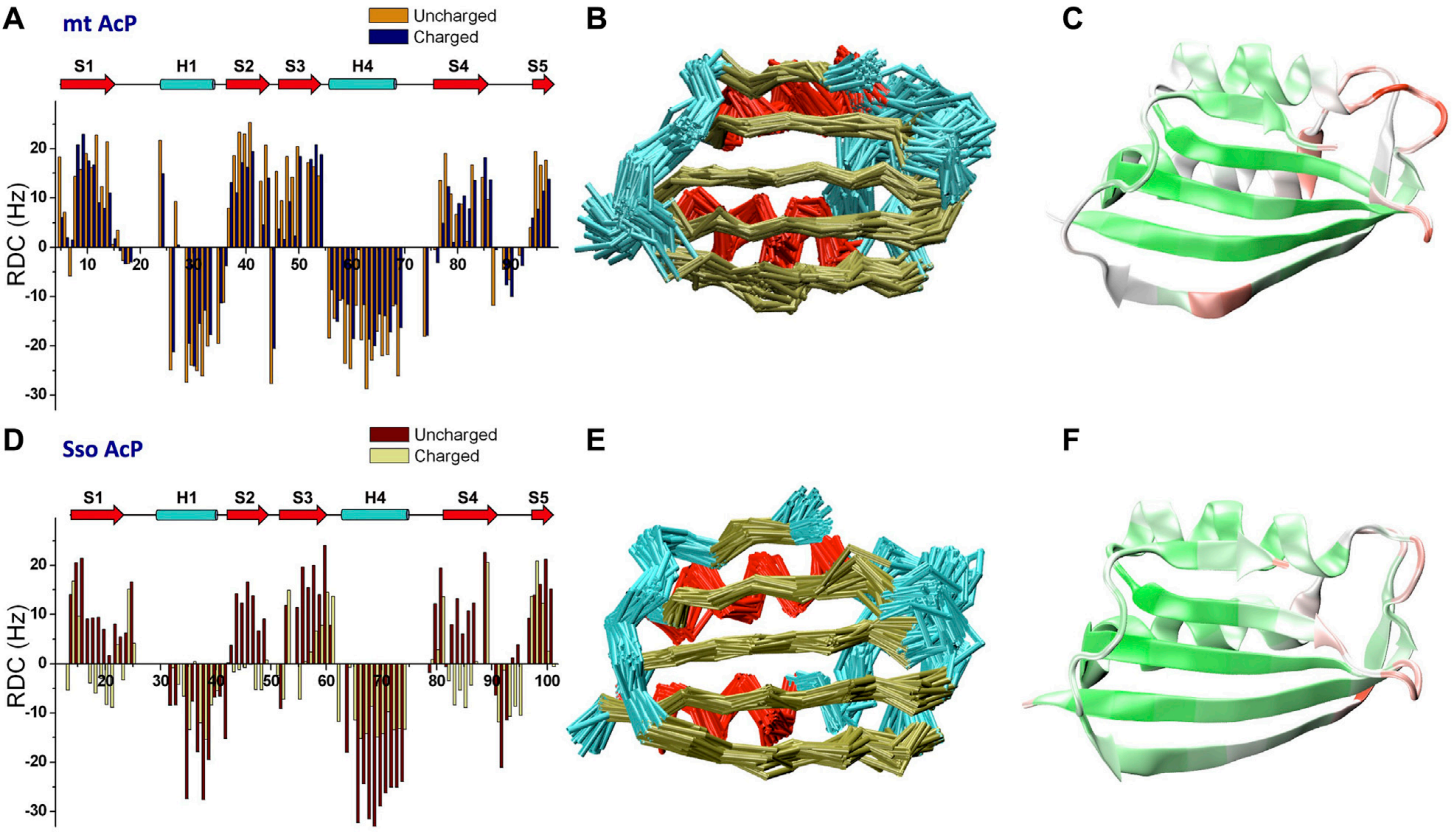
Microsecond dynamics from NMR residual dipolar couplings. **(A)** N-H RDCs measured for mt AcP at 37°C using charged and uncharged bicelles (Q factor 0.40). **(B)** Structural ensemble of mt AcP calculated using replica-averaged NMR-restrained MD incorporating 146 N-H and 192 C-N RDC values measured at 37°C. Red, gold and cyan colours for the Cα-traces indicate α-helixes, β-strands and loops, respectively. **(C)** RMSF values of the structural ensemble of mt AcP at 37°C plotted onto the structure. Color code range from low (green) to high (red) RMSF values. The corresponding RMSF graph is shown in Supplementary Figure S7. **(D)** N-H RDCs measured for Sso AcP at 80°C using charged and uncharged stretched gels (Q factor 0.68). **(E)** Structural ensemble of Sso AcP calculated using 144 N-H and 176 C-N RDC values measured at 80°C. Colour code as in panel B. **(F)** RMSF values of the structural ensemble of Sso AcP at 80°C plotted onto the structure. Colour code as in panel C. The corresponding RMSF graph is shown in Supplementary Figure S7.

RDCs are powerful NMR observables to probe protein structure and fluctuations in the μs timescale (Tjandra and Bax, 1997; Mukrasch et al., 2007; Lange et al., 2008; De Simone et al., 2009; Montalvao et al., 2012; De Simone et al., 2015), a window of protein motions that is relevant for key biomolecular processes such as catalysis, allosteric regulation and protein-protein interactions (Boehr et al., 2006a). We here measured NMR RDC using a spectrometer operating at the ^1^H frequency of 700 MHz. RDC data for mt AcP at 37°C and Sso AcP 80°C were acquired using both neutral and charged alignment media. For mt AcP, RDC were measured using DMPC/DHCP/CTAB bicelles in positively charged (ratio 2.9:1.0:0.2) and uncharged (ratio 2.9:1.0:0) modes. A total of 146 N-H RDC values were collected using ^1^H-^15^N HSQC spectra in IPAP mode and across the two alignment media (Figure 2A), showing relatively different alignment tensors (Q factor of 0.4, Supplementary Figure S4). The analysis of the RDCs of mt AcP at 37°C identified distinctive patterns of secondary structure elements, with helices and strands showing negative and positive RDC values, respectively (Figure 2A). In order to measure RDCs in regions where ^1^H resonances are broadened as a result of the conformational exchange (Supplementary Figure S2), we employed ^13^C-detected CON (Bermel et al., 2003; Hsu et al., 2009; Schiavina et al., 2019), providing additional 192 C-N RDCs across the two alignment media (Supplementary Figure S5).

In the case of Sso AcP, we optimized a protocol to generate stable alignment media at 80°C for the duration of the RDC measurements (24h/48h). Since common alignment media such as bicelles, phages, membranes or liquid crystals are not effective at these temperatures, we used acrylamide gels and optimized the conditions of polymerization and stretching to obtain stable water splitting at this temperature. To prevent progressive loss of alignment due to evaporation of the solvent from the gel, we applied a drop of mineral oil also on top of the gel after stretching, leading to media that remained stable for the duration of the experiments. 144 N-H (Figure 2D) and 176 C-N (Supplementary Figure S5) RDC values of Sso AcP were measured using uncharged (5.11% w/v acrylamide, 0.13% bis-acrylamide, 0.1% ammonium persulfate and 0.0031% TEMED) and negatively charged (copolymerization 1:3 of acrylic acid and acrylamide) gels equilibrated in 30 mM MES buffer and pH 6.5. The data showed patterns of positive and negative RDCs, clearly identifying the secondary structure elements of the protein.

We then used RDCs to calculate accurate structural ensembles describing the conformational fluctuations of the two acylphosphatases at their respective physiological temperatures. Indeed, RDC-restrained MD simulations have proved to be powerful tools to describe the motions of proteins on the μs timescale (De Simone et al., 2011b; Montalvao et al., 2012; De Simone et al., 2013a; De Simone et al., 2013b; De Simone et al., 2015). The structural ensemble of mt AcP at 37°C was calculated using 146 N-H and 192 C-N RDC (Figure 2B). The ensemble showed significant agreement with the experimental RDCs (Supplementary Figure S6A-B), and described backbone fluctuations featuring significant dynamics for the loops connecting strand S1 and helix H1 (residues 18–22), strands S2 and S3 (residues 43–46) and helix H2 and strand S4 (residues 70–73) (Figure 2C). These regions are clustered around the active site, with the strongest fluctuations observed in the phosphate-binding loop (residues 18–22). This finding is in agreement with orthogonal ^15^N-relaxation experiments, where local high R_2_/R_1_ values indicated conformational exchange on slow timescales. In addition, a dynamical region in the ensemble of mt AcP was observed in the center of the β-strand S4, which is unusual for a secondary structure element. This region corresponds to a β-bulge that is conserved in strands S4 of the acylphosphatase family, and has been suggested to act as a negative design element against protein aggregation promoted by edge-to-edge intermolecular β-sheet formation (Richardson et al., 2002; Soldi et al., 2008), a mechanism involved in native-like protein aggregation (Plakoutsi et al., 2006; Bemporad et al., 2008). Our data clarify that the β-bulge disfavors the aggregation of mt AcP by an entropic cost associated with the significant levels of conformational dynamics in the strand S4.

**FIGURE 3:**
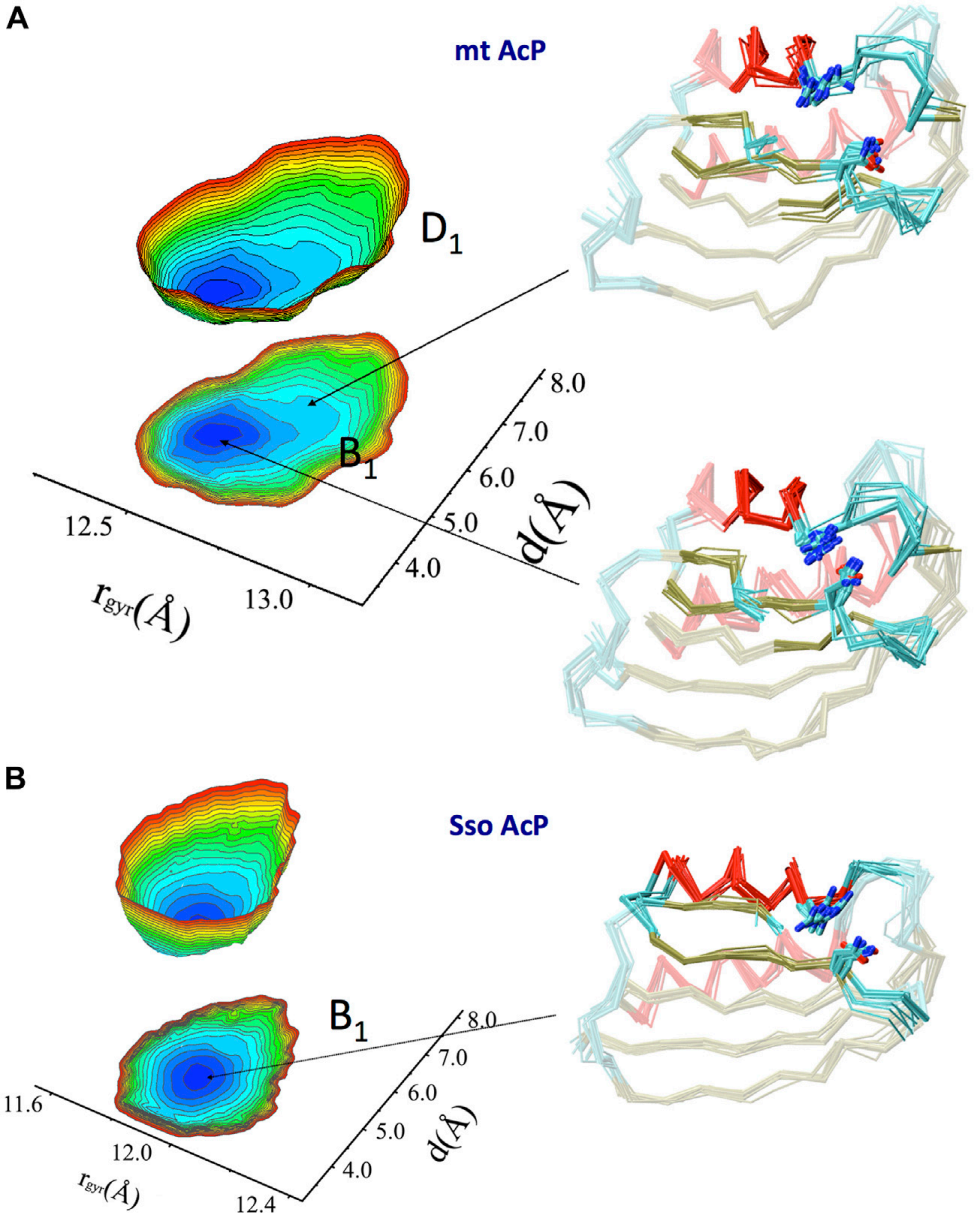
Native free energy surfaces of mt AcP and Sso AcP. **(A)** FES of mt AcP at 37°C calculated using RDC-derived structural ensemble projected onto two reaction coordinates. These are the protein radius of gyration (rgyr) of the entire protein (global coordinate) and the distance between the catalytic residues Arg 23 and Asn 41 (local coordinate). The FES is plotted either as 3D surface or projection onto the 2D plane. Representative structural bundles are shown for the main basin B1 and a distorted conformation D1 that is thermally accessible within the native state of the protein. Colour codes for the Cα-traces are as in Figure 2B. Arg 23 and Asn 41 are explicitly drawn on the protein ensemble. The free energy difference between the lowest (blue) and highest (red) points of the surface is 10 kJ/mol, whereas the free energy difference between the indicated B1 and D1 basins is 2.2 kJ/mol. **(B)** FES of SsoAcP at 80°C. Details as in panel A.

A Free energy surface (FES) was then obtained by projecting the RDC-restrained ensemble onto a global coordinate and one that is local to the active site (Figure 3A). These coordinates are the gyration radius of the protein (global) and the distance between side chains of the catalytic residues Arg 23 and Asn 41 (local). The FES identified a main conformational basin (B_1_) corresponding to a compact ground state featuring the catalytic residues in close proximity, and distorted conformations that are thermally accessible at 37°C (D_1_, free energy difference of 2.2 kJ/mol) and featuring an open active site with a generally less compact global protein shape. The landscape therefore indicates a conformational pathway between compact and expanded states, respectively with an inaccessible and accessible active site, where the binding affinity of the substrate is expected to be different.

Similarly to mt AcP, the structural ensemble of Sso AcP at 80°C was calculated using 144 N-H and 176 C-N RDC restraints (Figure 2E). The back-calculated RDCs from the structural ensemble were in excellent agreement with the experimental values (Figure S6). The ensemble identified the most dynamical regions of the backbone of Sso AcP, which were located in loops structurally clustered around the active site (Figure 2F), particularly in regions connecting strand S1 with helix H1 (residues 25–28) and strands S2 with S3 (residues 48–52). While this finding is in line with the analysis of mt AcP (Figure 2C), no correlations between global and local motions were found in the FES of Sso AcP (Figure 3B). In particular, Sso AcP at 80°C showed a deeper FES minimum (19 kJ/mol) compared to mt AcP (10 kJ/mol) with no thermally accessible distorted states. Another key difference in the dynamics of Sso AcP at 80°C and mt AcP at 37°C, is that the thermophilic protein has no dynamical regions in middle of the strand S4, suggesting that it has not been evolutionary optimized to employ this negative design element for avoiding edge-to-edge aggregation. RMSF values for the secondary structure elements also indicated higher rigidity for Sso AcP at 80°C (0.73 Å) than mt AcP at 37°C (0.80 Å, Supplementary Figure S7), which is in line with orthogonal S^2^ from ^15^N relaxation measurements (Figures 1C,D).

### Millisecond dynamics by NMR HX.

**FIGURE 4:**
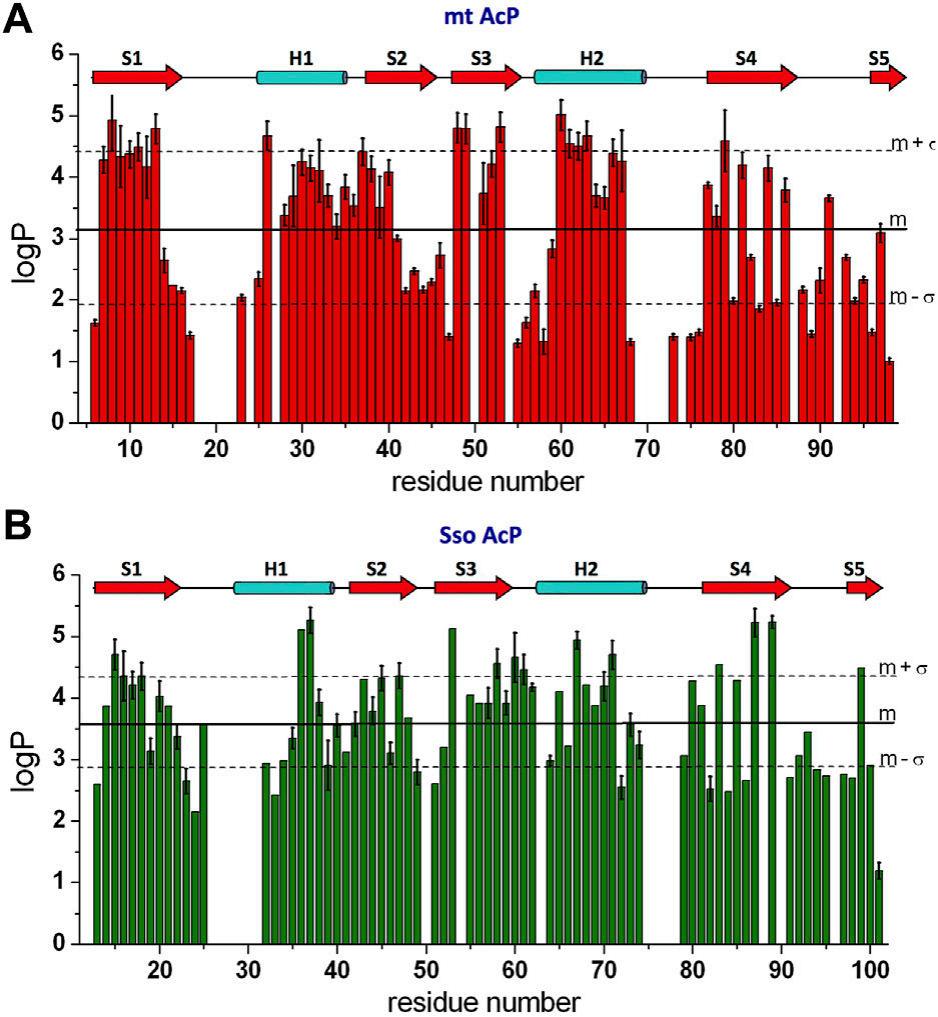
Millisecond dynamics from NMR HX experiments. **(A)** NMR HX data of mt AcP at 37°C elaborated as logP values. Error bars are derived from the fitting standard deviation of the raw exchange NMR data. **(B)** NMR HX data of Sso AcP at 80°C.

HX is a direct probe of protein dynamics on the millisecond timescale (and beyond). In order to compare the slow dynamics in mt AcP and Sso AcP at their physiological temperatures, we measured HX via NMR using a spectrometer operating at the ^1^H frequency of 700 MHz. *k*_*obs*_ values for the backbone amide HX were directly determined from the attenuation of the signals in ^1^H-^15^N HSQC spectra upon dissolving lyophilised protonated protein in D_2_O buffered solutions. These measurements detected 56 HX *k*_*obs*_ for amide backbone atoms of mt AcP at 37°C in 30 mM MOPS buffer, pD 7.0. As 20 amides exchanged completely prior to acquisition of the first ^1^H-^15^N HSQC spectrum of mt AcP under these conditions, for these residues we used CLEANEX experiments (Hwang et al., 1998) to evaluate fast H-H exchange as previously used with other AcP enzymes (De Simone et al., 2011a) as well as hyperthermophilic proteins (Hernandez et al., 2000), therefore determining 21 additional *k*_*obs*_ values. The *k*_*obs*_ values were normalised with theoretical values calculated from the sequence (Connelly et al., 1993) and assuming that the peptide chain is in a random coil conformation (*k*_*int*_) to obtain the protection factor logP from the logarithm of *k*_*int*_/*k*_*obs*_. Overall the measured pattern of logP values along the sequence showed well-protected secondary structure elements as well as exposed regions vulnerable to amide hydrogen exchange with water (Figure 4A). Moreover, consistently with the protein topology, edge β-strands S4 and S5 were found to be associated with alternating patterns of amide protection (Figure 4A).

HX *k*_*obs*_ values of Sso AcP at 80°C were directly measured for 56 amide backbone atoms by observing the decay in the peak intensities in the ^1^H-^15^N HSQC spectra of Sso AcP as a function of time after dilution into deuterated MES buffer (30 mM) at pD 6.5. Additional 15 *k*_*obs*_ values for residues that exchanged completely prior to acquisition of the first ^1^H-^15^N HSQC spectrum were obtained with CLEANEX experiments (Hwang et al., 1998). As in the case of mt AcP, the logP values along the sequence identified the pattern of protected secondary structure elements as well as unprotected amides that are exposed to the solvent (Figure 4B).

Overall, the comparison of the HX data of mt AcP and Sso AcP shows that the level of amide protection of the two proteins is comparable with average logP values of 3.63 ± 0.85 for Sso AcP at 80°C and 3.11 ± 1.20 for mt AcP at 37°C. This finding adds to previous FT/IR investigations obtained for other proteins (Zavodszky et al., 1998), and offers an in-depth inspection of HX at a residue specific resolution. In addition to the observed consistency in the patterns of high LogP for secondary structure elements, an interesting similarity between the two AcPs was found in highly unprotected loop regions, amounting to 21% of the structured domains of the two proteins excluding the N- and C- terminal tails (i.e. starting at strand S1 and ending at strand S5). Thus, in contrast to the general idea that thermophilic proteins have evolved shorter solvent-exposed loops (Balasco et al., 2013; Ruggiero et al., 2019), mt AcP and Sso AcP were here found to have similar amounts of water accessible regions.

## 4 Discussion

The intriguing characteristic of proteins and enzymes from thermophile organisms is that they possess biological function under conditions that inactivate or unfold their mesophilic homologues (Holliday et al., 2015; Mangiagalli et al., 2021). The adaptation of life at high temperature has been achieved via many strategies, including the use of molecular chaperones (Liberek et al., 2008; Roodveldt et al., 2009) and degradation machineries (Molinari, 2007) to assist or clear misfolded proteins (Behrends et al., 2006; Bukau et al., 2006). But the primary route of thermal adaptation is encoded in intrinsic barriers in the conformational free energy of hyperthermophilic proteins that preserve the correct fold, solubility and biological activity at extremely high temperatures. A fundamental element of adaptation of these systems includes a fine “thermal tuning” that enables the optimal structural dynamics to be attained at high temperatures. Understanding the details of such optimisation is a crucial challenge in the quest of clarifying the general structure-function-activity relationship in protein molecules. Some studies have employed experimental techniques to probe protein dynamics at high temperature in fast (Miletti et al., 2015; Yang et al., 2022) or slow (Zavodszky et al., 1998; Butterwick and Palmer, 2006) dynamical regimes. MD simulations have also been extensively exploited in this research area (Stafford et al., 2013; Katava et al., 2016; Stirnemann and Sterpone, 2017; Maffucci et al., 2020), however, it might be argued whether the simplifications in the empirical force fields represent critical limitations to elucidate the fine-tuning of protein dynamics at different temperatures.

In order to achieve a new level of understanding of the role of functional structural dynamics in the thermal adaptation of thermophilic proteins, we measured RDC data and used them to generate accurate structural ensembles of two homologous AcP enzymes whose respective physiological temperatures differ by 43°C. As RDCs are accurate probes of microsecond fluctuations in proteins, a fundamental dynamic regime for enzymatic activity (Boehr et al., 2006a), we complemented these data with ^15^N-relaxation and HX NMR to achieve a holistic map of timescales collectively ranging from nanoseconds to milliseconds (and beyond). On the nanosecond timescale, ^15^N-relaxation experiments showed that, while preserving similar patterns of rigid and dynamical elements, the dynamics of mt AcP at 37°C and Sso AcP at 80°C differ with respect to regions that are in conformational exchange (high R_2_/R_1_ values). In addition, order parameters from model free analyses were found to be generally higher in Sso AcP, indicating that the *Sulfolobus solfataricus* AcP is more rigid at 80°C than its human homologue at 37°C. On the millisecond timescale, HX experiments generated a map of the protection factors at a residue-specific resolution of the two AcPs, indicating similar protection of backbone amide groups despite the very different physiological temperatures. Moreover, in contrast with the general idea that hyperthermophilic proteins feature shorter solvent-exposed loop regions than their mesophilic counterparts, a similar amount of regions that are poorly-protected from water exchange was observed in the two proteins.

The study of the microsecond dynamics based on RDC-restrained MD simulations identified distinctive elements of individual strategies of thermal adaptation in the two proteins. In particular, the ensemble of mt AcP at 37°C featured a dynamical segment in the centre of the edge strand S4, indicating a negatively designed element that exploits entropic energy barriers to disfavour edge-to-edge aggregation. The absence of such an element in Sso AcP at 80°C indicates a lack of specific strategies to reduce the aggregation propensity of strand S4. This finding can be due to the different evolutionary pressures in *Sulfolobus solfataricus* and in eukaryotes, possibly associated with diverse needs in preventing aggregation at the respective physiological temperatures, as suggested by proteomic studies (Leuenberger et al., 2017). Indeed the high kinetic energy of the strand S4 of SsoAcP at 80°C can provide similarly high entropic energy barriers as a protection against aggregation. Another distinctive conformational property of mt AcP was found to be a coupling between global motions and local conformations of the active site. In particular, thermally accessible μs fluctuations in mt AcP were found to connect the compact ground state, featuring a tightly closed active site, to less-compact conformations having an open active site. This coupling of motions, which modulates the ligand affinity of the protein, was not observed in Sso AcP, showing a single free energy basin of compact structures with a tightly closed active site, thereby suggesting different strategies of substrate recognition and binding in the two homologous proteins.

In summary, by probing multiple timescales of backbone dynamics of AcP enzymes featuring very different physiological temperatures, we could clarify the native properties of homologue proteins from hyperthermophiles and mesophiles. Besides a general consistency in the dynamics of the two proteins, supporting the concept of corresponding states (Zavodszky et al., 1998; Radestock and Gohlke, 2011), atomic-resolution structural ensembles identified distinctive properties, including motions associated protein-protein interactions leading to undesired aggregation as well as physiological protein-ligand recognition and binding. Thus, despite the similarities in structure and dynamics at the respective physiological temperatures, mt AcP and Sso AcP appear to have been optimised under different selection rules, presumably due to a diverse balance between entropic and enthalpic terms guiding macromolecular interactions. The ability to characterise the fine-tuning between protein structure and dynamics is therefore key to advance our understanding of how proteins have evolved to be functional in their native environments and is pivotal to inform how changes in the primary sequences can induce loss/gain of function in protein design.

## Data Availability

The raw data supporting the conclusion of this article will be made available by the authors, without undue reservation.
