# Supplementary material for: The role of structural dynamics in the thermal adaptation of hyperthermophilic enzymes
Source: Front Mol Biosci. 2022 Sep 7;9:981312. doi: 10.3389/fmolb.2022.981312 (PMC9490001; doi:10.3389/fmolb.2022.981312)
Supplement: Supplementary file 1 [file DataSheet1.PDF]

frontiers

Supplementary Material

The Role of Structural Dynamics in the Thermal Adaptation of Hyperthermophilic Enzymes

Giuliana Fusco[1], Francesco Bemporad[2], Fabrizio Chiti[2], Christopher M. Dobson[1], Alfonso De Simone[3,4,*]

[1]Department of Chemistry, University of Cambridge, Lensfield Road, CB2 1EW, Cambridge UK

[2]Section of Biochemistry, Department of Experimental and Clinical Biomedical Sciences "Mario Serio", University of Florence, Viale GB Morgagni 50, 50134 Florence, Italy.

[3]Department of Life Sciences, Imperial College London, South Kensington, SW7 2AZ UK

[4]Department of Pharmacy, University of Naples "Federico II", via Montesano 49, 80131, Naples Italy

*** Correspondence:**
Alfonso De Simone
alfonso.desimone@unina.it

Figures S1-S7

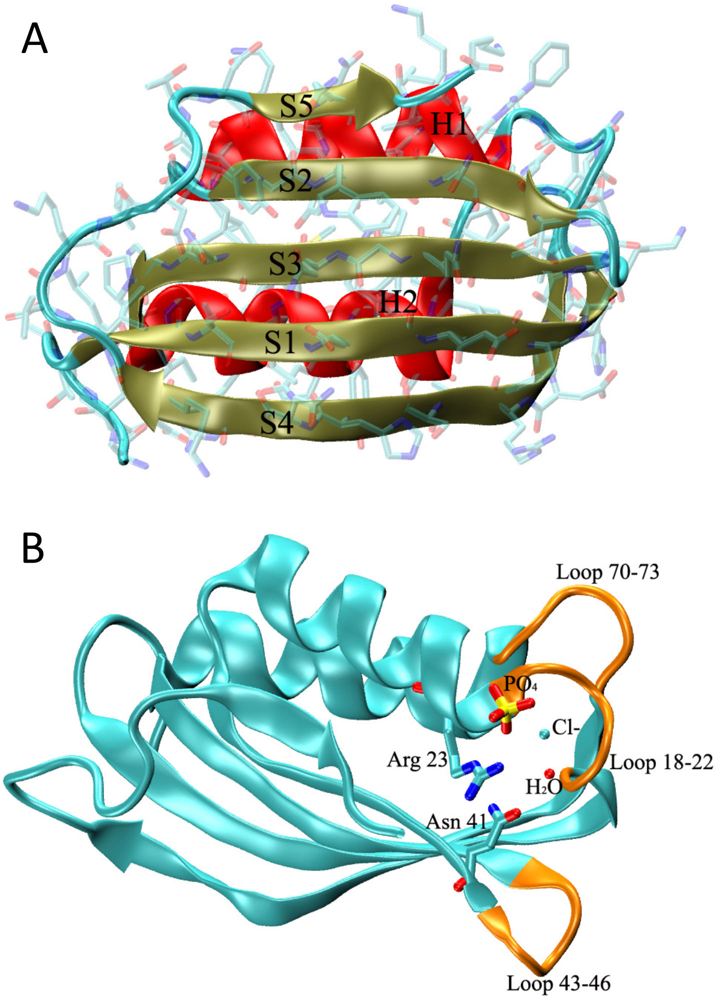

Figure S1. Crystal structure of common-type acylphosphatase determined at 1.8 Å resolution. PDB code: 2acy. **A**) General topology of acylphosphatases. Strands S1-S5 are colored in gold, helices H1 and H2 are colored in red whereas loops are colored in cyan. **B**) Active site of common-type acylphosphatase bound to chloride and sulfate ions.

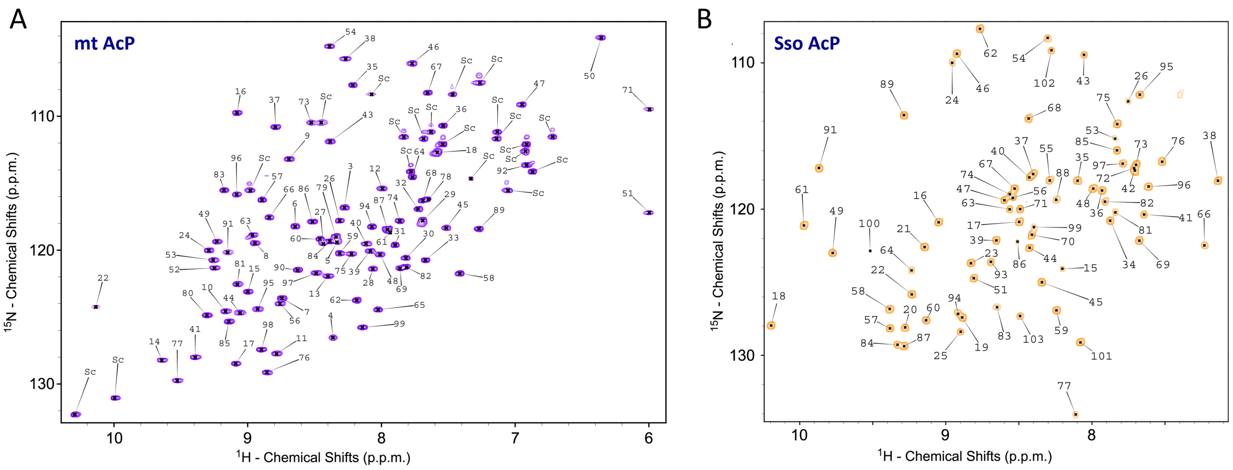

Figure S2. Assigned 2D ^{1}H-^{15}N HSQC spectra. A) ^{1}H-^{15}N HSQC spectrum of mt AcP at 37 °C. All the spectrum resonances were assigned. Missing resonances that are broadened beyond detections are associated with residues 1-2, 19-21, 23, 25, 42, 70, 88 and 93. **B**) ^{1}H-^{15}N HSQC spectrum of Sso AcP at 80 °C. All the spectrum resonances were assigned. Missing resonances that are broadened beyond detections are associated with residues 12-14, 27-33, 65, 80, 90, 92 and 98.

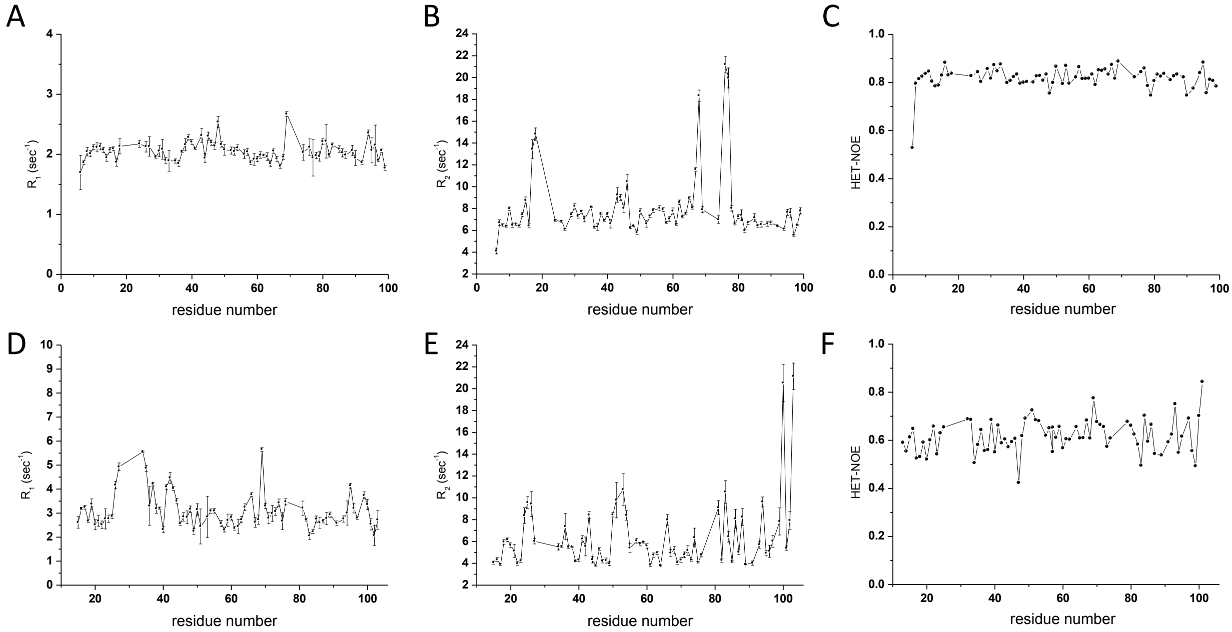

Figure S3. ^{15}N relaxation experiments. A-C) Data for mt AcP at 37 °C, including R_1 values (A) R_2 values (B) and hetNOEs (C). **D-F**) Data for Sso AcP at 80 °C, including R_1 values (D) R_2 values (E) and hetNOEs (F).

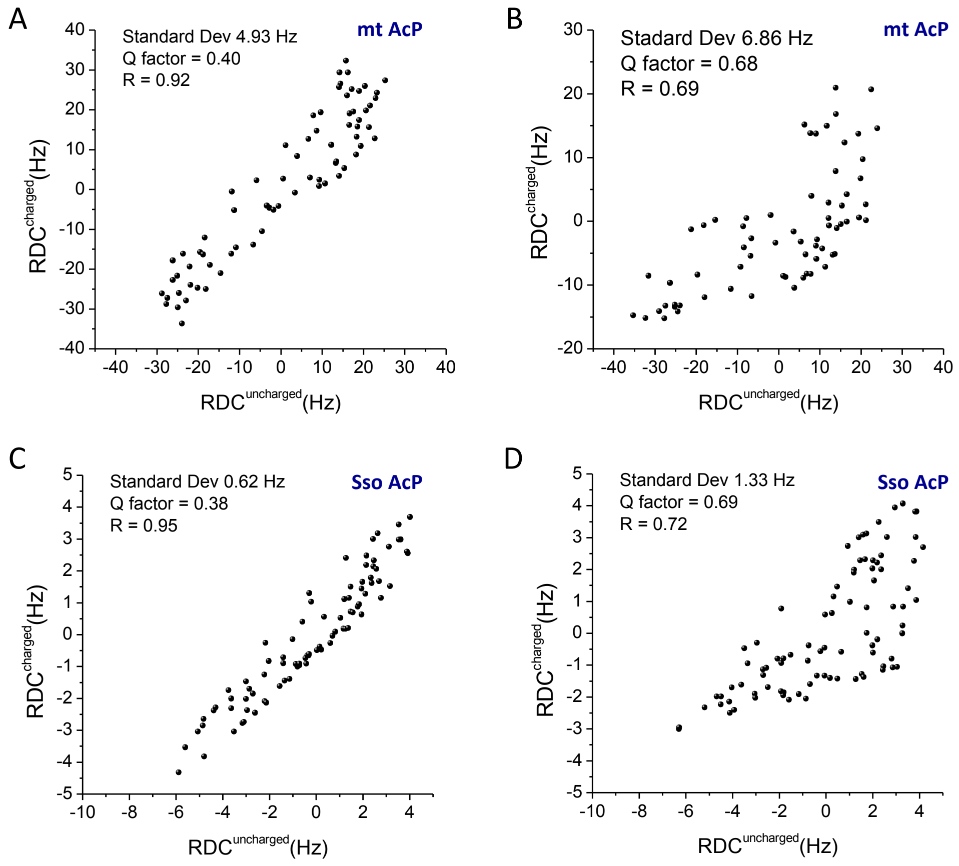

Figure S4. Scatter plots of N-H and C-N RDCs. A-B) N-H (A) and C-N (B) RDCs for mt AcP measured using charged and uncharged bicelles at 37 °C. **C-D**) N-H (C) and C-N (D) RDCs for Sso AcP measured using charged and uncharged gels at 80 °C.

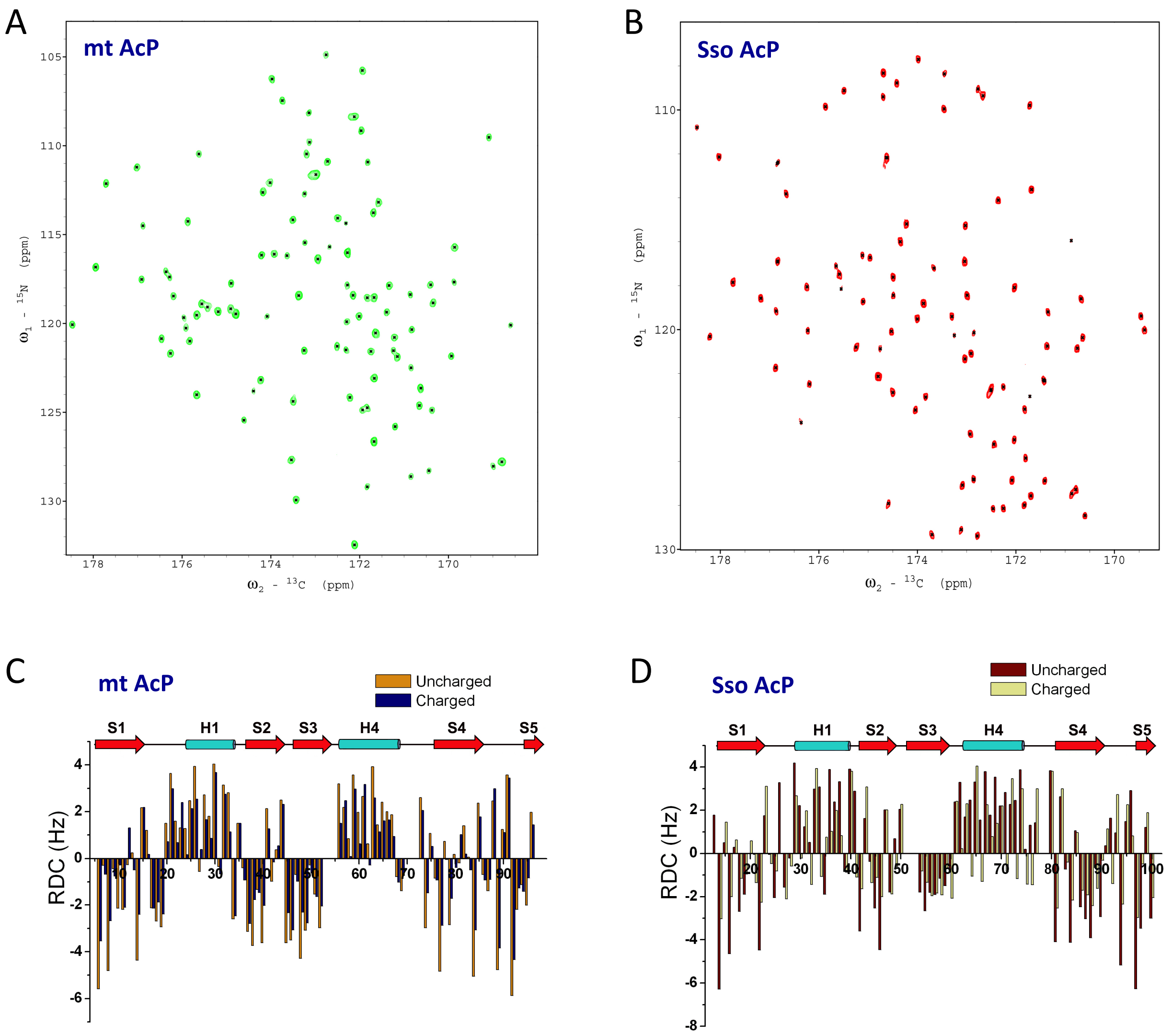

Figure S5. N-C RDCs measured using ^{13}C-detected CON. A-B) CON experiments for mt AcP at 37 °C (A) and Sso AcP at 80 °C (B). **C**) N-C RDCs for mt AcP measured using charged and uncharged bicelles at 37 °C (Q factor 0.38). **D**) N-C RDCs for Sso AcP measured using charged and uncharged gels at 80 °C (Q factor 0.69).

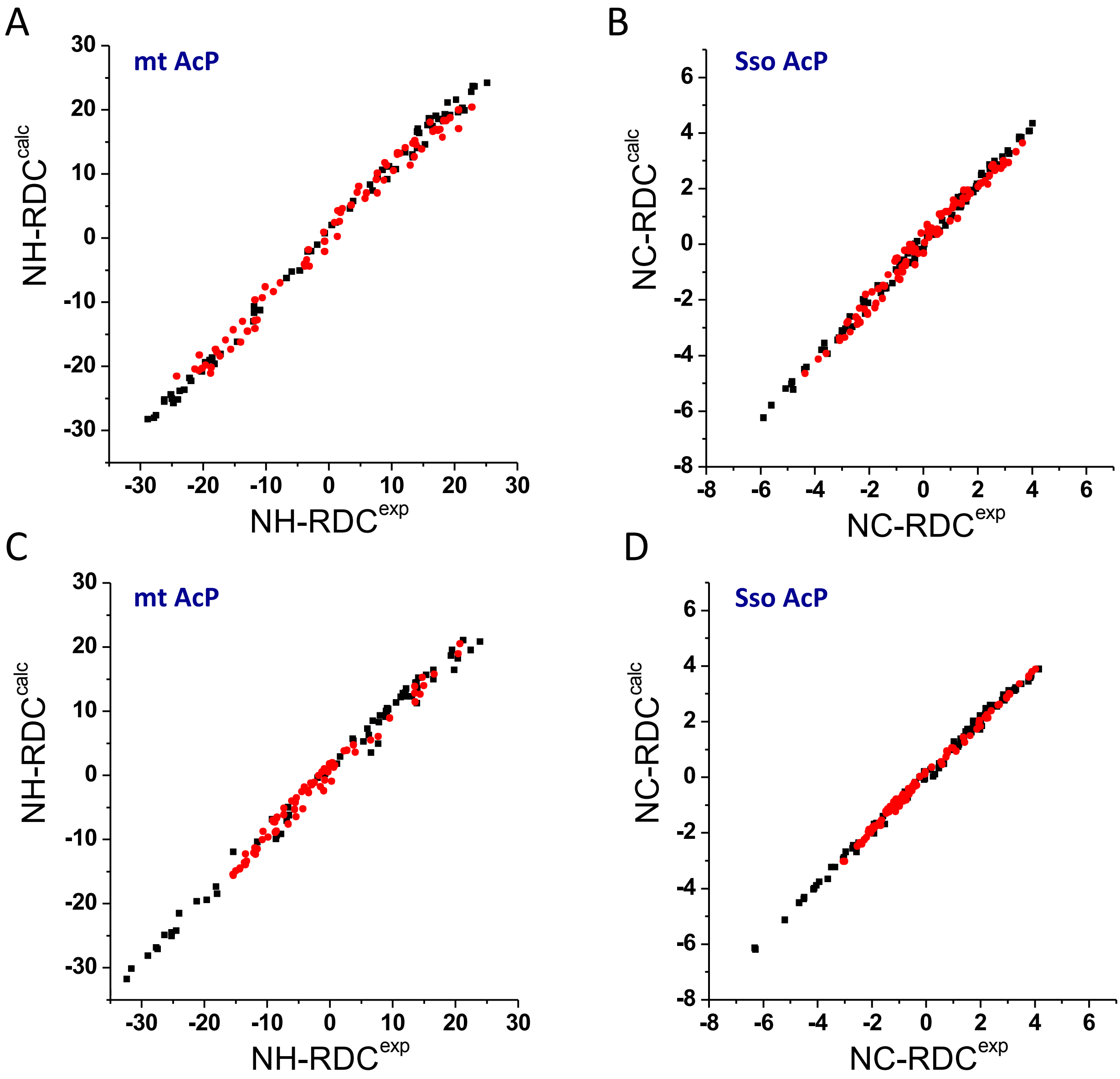

Figure S6. Agreement between experimental RDCs and those calculated from the structural ensembles. Black and red points indicate RDCs measured with uncharged and charged alignment media. **A-B**) N-H (A) and N-C (B) RDCs for mt AcP at 37 °C. The Q factors between experimental and calculated data are 0.16 and 0.15, respectively. **C-D**) N-H (C) and N-C (D) RDCs for Sso AcP at 80 °C. The Q factors between experimental and calculated data are 0.15 and 0.14, respectively.

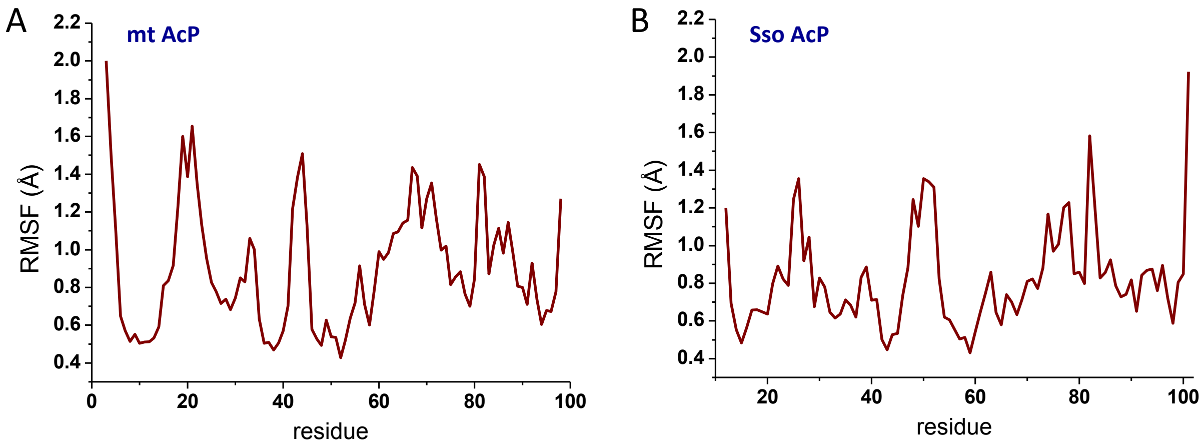

Figure S7. Root mean square fluctuations (RMSF) calculated from the structural ensembles. A) mt AcP at 37 °C. **B**) Sso AcP at 80 °C.
